# The challenging interplay between rheumatoid arthritis, ageing and comorbidities

**DOI:** 10.1186/s12891-016-1038-3

**Published:** 2016-04-26

**Authors:** Marloes van Onna, Annelies Boonen

**Affiliations:** Department of Internal Medicine, Division of Rheumatology, Maastricht University Medical Center, School for Public Health and Primary Care (CAPHRI), Maastricht University, P. Debyelaan 25, Maastricht, 6202 AZ The Netherlands

**Keywords:** Rheumatoid arthritis, Ageing, Elderly, Comorbidity, Multimorbidity

## Abstract

**Background:**

The incidence of rheumatoid arthritis (RA) is expected to increase over the next 10 years in the European Union because of the increasing proportion of elderly people. As both RA and ageing are associated with emerging comorbidities such as cardiovascular disease, malignancies and osteoporosis, these factors will have a profound effect on the management of RA. In addition, both increasing age and comorbidities may independently alter commonly used RA-specific outcome measures.

**Discussion:**

Age-related decline in immune cell functions (immunosenescence), such as a decrease in T-cell function, may contribute to the development of RA, as well as comorbidity. The chronic immune stimulation that occurs in RA may also lead to premature ageing and comorbidity. The interplay between RA, ageing and (emerging) comorbidities is interesting but complex. Cardiovascular disease, lung disease, malignancies, bone and muscle wasting and neuropsychiatric disease all occur more frequently in RA patients as compared to the general population. It is unclear how RA should be managed in ‘today’s world of multiple comorbidities’. Evidence that treatment of RA improves comorbidities is currently lacking, although some promising indirect observations are available. On the other hand, there is limited evidence that medication regularly prescribed for comorbidities, such as statins, might improve RA disease activity. Both ageing and comorbidity have an independent effect on commonly used outcome measures in the RA field, such as the Health Assessment Questionnaire (HAQ) and the clinical disease activity index (CDAI). Prospective studies, that also account for the presence of comorbidity in (elderly) RA patients are therefore urgently needed. To address gaps in knowledge, future research should focus on the complex interdependencies between RA, ageing and comorbidity. In addition, these findings should be integrated into daily clinical practice by developing and testing integrated and coordinated health care services. Adaptation of management recommendations is likely required.

**Summary:**

The elderly RA patient who also deals with (emerging) comorbidities presents a unique challenge to treating clinicians. A paradigm shift from disease-centered to goal-oriented approach is needed to develop adequate health care services for these patients.

## Background

By 2030, about one in four inhabitants of the European Union will be above the age of 65 [1]. The relevance of ageing is becoming more and more apparent in industrialized countries as, in parallel to an increase in life expectancy, birth rates are decreasing [1]. In an ageing population, it is expected that the number of patients with inflammatory arthritis, including rheumatoid arthritis (RA), will grow proportionally. RA is known to have a high disease burden and is associated with a substantial economic burden on patients, their families, and society [2, 3]. It is estimated that in England the annual direct healthcare costs of RA are approximately €780 million per year and the indirect costs related to work disability up to €6.75 billion per year [4]. A considerable proportion of these costs is due to the fact that RA is a complex disease associated with an increased prevalence of several comorbidities [5, 6]. These comorbidities can precede or accompany RA, and can be caused by the therapeutic armamentarium used in patients with RA. Substantial evidence indicates that the continuous systemic inflammation and immune dysfunction characteristic for RA plays a critical role in the development and acceleration of comorbidities [7]. Comorbidities most frequently seen in patients with RA include cardiovascular disease, lung disease, malignancies, osteoporosis, changes in body composition and neuropsychiatric disease. Most of these comorbidities occur more frequently than expected in RA patients as compared to the general population. As the number of comorbidities increase with age, and as patients with RA survive longer, more patients with RA will have comorbidities. Currently, the average patient with RA has two or more comorbid disorders [6, 8, 9].

Resolving the interplay between RA, comorbidities and its determinants is challenging. While the occurrence of (emerging) comorbidities is more common in RA, the clinical consequences of comorbidity are also more severe in these patients as compared to controls. Despite this observation, comorbidity is often underrecognized and undertreated [6, 10, 11]. Many guidelines and outcome measures for RA focus on RA as a single disease and disregard that presence of comorbidity is nowadays the rule and not the exception.

**Fig. 1:**
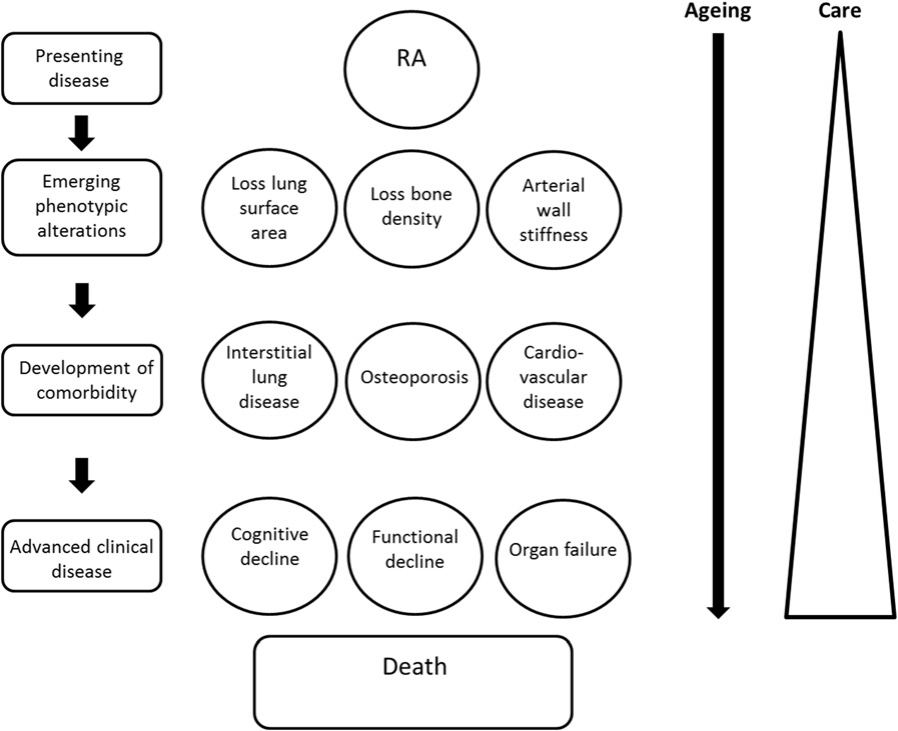
Conceptual model for the effects of ageing and development of comorbidity in patients with RA. Abbreviation: RA, rheumatoid arthritis

Future research is therefore urgently needed. However, to facilitate the identification of knowledge gaps, it is important to reflect on what is currently known. This narrative overview will first address the process of ageing and immuno-senescence in patients with RA. Next, existing data on the role of RA or its management on the occurrence or course of comorbidities is summarized. In the following part, literature on implications of ageing and comorbidities on outcome assessment and management of RA is presented. If available, results of meta-analyses or systematic reviews are presented. In the last part, it is shown how treatment for RA may positively influence comorbidity and how treatment of comorbidity may positively influence RA. We will show that research addressing this topic has been scattered across multiple disciplines and a solid evidence base upon which to build policy is currently lacking. To keep up with the continuing demographic shift of an ageing (RA) population, we need to expand our knowledge on ageing and emerging comorbidities in patients with RA in order to develop adequate health care services for these patients (Fig. 1).

## Discussion

### Rheumatoid arthritis, ageing and immunosenescence

**Fig. 2:**
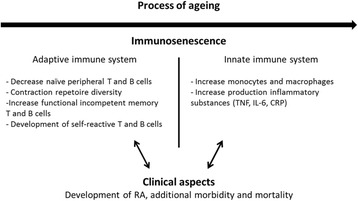
The interplay between immunosenescence and development of rheumatoid arthritis [15–17]. Abbreviations: RA, rheumatoid arthritis; TNF, Tumor Necrosis Factor; IL-6, Interleukin-6; CRP, C-reactive protein

To understand how ageing may affect RA and vice versa, one should first understand the contribution of the underlying cellular mechanisms. Senescence is a normal biological process that occurs in all organisms and involves the age-related decline in cell functions. In the context of the ageing immune system, this phenomenon is known as immunosenescence [12]. The mechanisms behind this process are multidimensional, but key features include age-related changes in both the adaptive and innate immune system. Immunosenescence of the adaptive immune system is characterized by loss of regenerative capacity and defects in T and B cell production, maturation and function (Fig. 2) [12–15]. Most profoundly, the ability to activate T cells in a productive manner is decreased. Because of the absence of an adequate T cell activation, the differentiation and effector function of B cells is also hindered [15, 16].

In addition to loss of effectiveness of the adaptive immune system, immunosenescence is also characterized by an enhanced chance to develop autoimmune disorders, including RA [16, 17]. The co-occurrence of declining immunocompetence and increasing autoimmune susceptibility appears contradictory at first sight. There are however parallels. It is suggested that the lack of immune system stability predisposes to tolerance failure [12, 13]. For instance, CD28 deficiency in CD4 T-cells is associated with an increased production of proinflammatory cytokines [14]. In addition, alterations in the innate immune system cause monocyte and subsequent macrophage activation, resulting in an increase in levels of Tumor Necrosis Factor (TNF), Interleukin-6 (IL-6), C-reactive protein (CRP) and other inflammatory substances (Fig. 2) [12, 17]. This proinflammatory environment may, together with the development of self-reactive T and B cells, promote the development of RA. Alternatively, the continuous systemic inflammation in established RA may induce accelerated immunosenescence and development of other morbidities, such as cardiovascular disease (CVD) and cachexia (Fig. 2) [12, 17, 18].

## Rheumatoid arthritis and presence of comorbidity

### Cardiovascular disease

CVD usually encompasses coronary heart disease, peripheral vascular disease, cerebrovascular disease and congestive heart failure. It might also include prognostic markers of disease such as hypertension or dyslipidemia. The association of RA with accelerated atherosclerosis and eventually cardiovascular disease is a well-established one. The combined risk of cardiovascular morbidity is doubled in RA patients and there is a 60 % increase in risk of cardiovascular mortality [19–23]. In a meta-analysis of 14 observational studies including 41.490 RA patients by Avina-Zubieta et al., the risk of a myocardial infarction and cerebrovascular accident were increased by almost 70 % (pooled Relative Risk (RR) 1.7 (95 %-CI 1.4-2.0)) and 41 % (pooled RR 1.4 (95 %-CI 1.1-1.7)), respectively [20].

The RA-associated increased risk of cardiovascular morbidity and mortality can be explained by several processes that often occur simultaneously: (1) the effect of RA itself due to the presence of chronic systemic inflammation, (2) the effect that the presence of RA modulates important traditional cardiovascular risk factors or (3) the use of RA-specific medication such as non-steroidal anti-inflammatory drugs (NSAIDs), corticosteroids and disease modifying anti-rheumatic drugs (DMARDs) [5]. Smoking, hypertension, insulin resistance, physical inactivity, dyslipidaemia and obesity are highly prevalent in people with RA as compared to the general population [24]. A recent meta-analysis suggested that the risk of cardiovascular events is increased when patients with RA frequently use corticosteroids ((RR) 1.5; 95 %-CI 1.3-1.6; p < 0.001) and NSAIDs (RR 1.2; 95 %-CI 1.0-1.4; p = 0.04) [25]. Interestingly, in a study by Lindhardsen et al., based on approximately 10 000 patients with RA, the risk of a myocardial infarction in patients with RA was similar to the risk of MI in patients without RA who were 10 years older [26].

### Lung disease

Interstitial lung disease (ILD) and pleuritis are one of the most common extra-articular manifestations of RA [27]. In addition, lung disease may also be related to drug therapy used in RA or related to other comorbid disorders. ILD is the most important pulmonary manifestation of RA. Bongartz et al. found in a longitudinal study of 582 RA patients and 603 non-RA individuals, that the lifetime risk of developing ILD was 7.7 % for RA patients and 0.9 % for non-RA individuals (HR 9.0 (95 %-CI 4.0-19.9)) [28]. The association between Chronic Obstructive Pulmonary Disease (COPD), and RA is less well established [29]. In a meta-analysis of Ungprasert et al., that included 4 retrospective cohort studies (32.675 RA patients and 122.204 controls), the pooled RR of incident COPD in RA patients versus controls was 2.0 (95 %-CI 1.6-2.5) [29]. However, confounding might be responsible for the association between COPD and RA as smoking is also a well-established risk factor for RA [29].

### Malignancies

In a meta-analysis including a total of 21 studies, RA was an independent risk factor for the development of lymphoma and was associated with a lymphoma risk that is approximately two-fold increased (standardized incidence ratio (SIR) 2.1, 95 %-CI 1.8-2.4) [30]. The risk on lymphoma appears to be especially higher in patients with high RA disease activity and presence of rheumatoid factor [31, 32]. There seemed to be a decreased risk for colorectal cancer (SIR 0.8, 95 %-CI 0.7-0.9) [30]. In a retrospective population cohort study among 84.475 RA patients who were observed for 405.540 person-years, it was found that RA patients had a significant higher risk of developing lung (SIR 1.7, 95 %-CI 1.5-1.8), liver (SIR 1.9, 95 %-CI 1.3-2.6), and oesophageal cancer (SIR 1.8, 95 %-CI 1.2-2.5), but a lower risk of prostate (SIR 0.7, 95 %-CI 0.6-0.7), breast (SIR 0.6, 95 %-CI 0.6-0.7) and ovarian cancer (SIR 0.6, 95 %-CI 0.5-0.8) cancer [33].

A recent systematic review of 49 studies showed that RA patients who used a TNF-inhibitor did not have an additional increased risk for malignancies in general, nor for lymphoma or non-melanoma skin cancer as compared to RA patients who did not use a TNF-inhibitor. However, the risk of melanoma might be increased (adjusted Hazard Ratio (HR) 1.5 (95 % CI 1.0-2.2)) [34].

### Osteoporosis and changes in body composition

Another important group of RA associated comorbidities include bone and muscle wasting. Osteoporosis is characterized by a decline in bone mineral density (BMD), which may eventually increase the chance of developing fragility fractures [35]. The lifetime fracture risk of a patient with osteoporosis is as high as 40 % [35]. In a study that included more than 30.000 RA patients selected from the British General Practice Research Database, the RR of a hip fracture was 2.0 (95 %-CI 1.8-2.3) and 2.4 (95 %-CI 2.0-2.8) for a vertebral fracture [36].

Cachexia due to systemic inflammation is characterized as the involuntary reduction in lean body mass (LBM) while fat mass tends to be maintained or even increased so that the body mass index (BMI) remains stable [37–40]. It has been shown that low LBM and higher fat mass is associated with a low BMD, even after controlling for potential confounders such as age, race, sex, height and grip strength [41, 42]. Specifically, He et al. showed among 17.891 individuals, that those with sarcopenia were two times more likely to have osteopenia or osteoporosis as compared to subjects without sarcopenia (OR 2.0; 95 %-CI = 1.6-2.6) [42]. The prevalence of cachexia in RA patients highly varies between patient populations and prevalence rates between 26-71 % have been reported [39]. The increase in fat mass is suggested to be a risk factor for the development of the metabolic syndrome and cardiovascular disease [40]. However, there are currently no studies that analysed cachexia in relation to cardiovascular mortality in RA.

### Cognitive impairment, depression and anxiety

A few small-size studies have evaluated the impact of cognitive impairment in patients with RA [43–46]. These studies suggest that cognitive impairment is more frequently observed in patients with RA as compared to controls [43, 44]. In one long-term population-based study of Wallin et al., RA in midlife was associated with cognitive impairment two decades later, even when correcting for concomitant cardiovascular disease (OR (95 %-CI): 2.7 (1.2–6.1)) [47]. In another study that did not include a control group, an impairment in visual-spatial tasks was detected in 71 % of 30 included patients [44]. Cognitive impairment is also associated with more functional limitations, pain and depression in patients with RA [46, 48]. Potential risk factors for cognitive impairment are educational level, income, oral glucocorticoid use and presence of CVD risk factors [45].

Depression and anxiety are highly prevalent in patients with RA and are associated with poorer RA outcomes [49]. In the United States, the 12-months prevalence estimates of depression and anxiety disorders in the general population were 6.6 % and 18.1 %, respectively [50, 51]. Especially the prevalence of depression is considerably higher in RA patients. In a meta-analysis that included 13.189 patients with RA from 72 studies, the prevalence of a major depressive disorder was found to be 16.8 % (95 %-CI 10 %-24 %) [52]. The prevalence of anxiety disorders in patients with RA varied between studies from 13 % to 22 % [53, 54].

### Effect of ageing and comorbidities on RA-specific outcome measures

The functional status of a patient with RA and response to treatment is measured by several disease-specific outcome measures, such as the Disease Activity Score −28 (DAS28), Health Assessment Questionnaire (HAQ) and the American College of Rheumatology (ACR) remission criteria [55–57]. These outcome measures are often used in randomised controlled trials (RCTs). The inclusion of patients in these RCTs is however restricted by stringent criteria. Therefore, patients included in RCTs often not resemble the spectrum of patients treated in the ‘real world’, i.e. elderly patients who often face comorbidity and polypharmacy.

Notwithstanding, several studies suggest that both ageing and comorbidity may independently alter commonly used RA-specific outcome measures, including joint scores, remission and response criteria and functional disability assessments [58–66]. In a population study of Krishnan et al. among 1530 adults in Finland, 76 % reported some pain and 83 % reported less than perfect general health. The overall mean value of the Visual Analogue Scale (VAS) pain was 20 mm [63]. Ageing was an independent predictor for higher scores on both the pain VAS and global assessment VAS in this study [63]. Sokka et al. concluded that only 15 % of the general population > 50 years old meet all four ACR remission criteria [64]. This finding suggests that the current remission criteria may not accurately identify remission in elderly patients. Ranganath et al. evaluated 1584 RA patients in a prospective cohort study and found that increasing numbers of comorbidities were independently correlated with less improvement in the clinical disease activity index (CDAI) after initiation of anti-rheumatic treatment [58]. The improvement in CDAI was 3.9 units greater in patients with three or fewer comorbidities as compared with patients with nine or more comorbidities [58]. In a study that included 380 RA patients by Radner et al., it was concluded that increasing levels of comorbidities are associated with increasing levels of disability within each domain of the HAQ [66].

## Implications of ageing and comorbidity for treatment of RA

### Rheumatologists’ perspectives on elderly patients with RA and comorbidity

Nowadays, intensive anti-rheumatic treatment strategies that adhere to the treat-to-target principle are used to treat patients with RA [67]. Several studies have however described the phenomenon of ‘age bias’ when treating elderly patients with RA. Age bias may eventually result in initiation of less intensive treatment regimens [68, 69]. In a study by Kremers et al., younger patients with RA were significantly more likely to receive DMARDs at an early stage (HR per 10-year decrease in age 1.4; 95 %-CI 1.3-1.5) as compared to their older counterparts [68]. Even when the level of disease activity and number of comorbidities were comparable between younger and older patients, rheumatologists still preferred the less intensive treatment option in older patients [68].

By using data from the CORRONA registry Tutunctu et al. found that the percentage of younger RA patients who were on DMARD combination therapy (40.5 %) or on TNF-inhibitors (33.1 %) was considerably higher than that of older RA patients (30.9 % and 25.0 %, respectively; p < 0.001) [70].

### Modification of shared lifestyle risk factors

Smoking cessation, promoting physical activity and maintaining a healthy body weight are all pivotal steps to reduce both the prevalence and severity RA, several comorbidities (e.g. CVD) and to reduce overall mortality [71–73].

Cigarette smoking significantly increases the risk of developing RA [74, 75]. In a meta-analysis of observational studies, the OR to be diagnosed with RA in males with 20 or more pack-years of smoking was 2.3 (95 %-CI: 1.6-3.4) [74]. Although the exact mechanism behind this effect remains uncertain, the process of citrullination is considered to be an important factor for the development of RA in the anti-citrullinated protein antibody (ACPA)-positive patients [75]. Whether (cessation of) smoking influences the disease course in patients with RA remains controversial. There is no clear association between smoking and HAQ, DAS28, CRP or the erythrocyte sedimentation rate (ESR) [71, 76, 77]. In a meta-analysis that combined the radiographic data of six cohorts it was concluded that smoking was not an independent risk factor for radiological progression in RA, but that the effect was mediated via ACPA [78].

Regular exercise training in patients with RA is associated with improvement of and functional ability (e.g. aerobic fitness and muscle strength) without exacerbating disease activity [79–82].

Studies that address the association between body weight and disease activity show conflicting results and a high body mass index (BMI) has been correlated with both higher [83–85] and lower RA disease activity [86].

The European League Against Rheumatism (EULAR) has formulated recommendations about the need and timing of cardiovascular risk assessment in patients with RA [87]. In general, cardiovascular risk assessment should follow national guidelines (in general be performed annually) [6]. However, currently, no RA-specific management model is available for risk assessment and management of cardiovascular disease. According to the EULAR recommendations, cardiovascular risk prediction charts (e.g. Framingham Risk Score) should be multiplied by a factor of 1.5 in case two out of three of the following criteria are present: (1) disease duration > 10 years; (2) presence of rheumatoid factor or ACPA; (3) presence of extra-articular manifestations [87].

Unfortunately, up to our knowledge, there are at this moment no RCTs that assess the efficacy of antihypertensive agent or statins on cardiovascular endpoints exclusively in RA patients.

### Does treatment of RA improve comorbidities?

The possible beneficial effects of anti-rheumatic treatment on concomitant cardiovascular disease has not been addressed in prospective RCTs. As mentioned before, patients with comorbidities are in fact often excluded from these RCTs. Most research concentrates on the question whether anti-rheumatic therapy may prevent the occurrence of cardiovascular events. Since both RA and atherosclerosis are inflammatory diseases, anti-rheumatic therapy may also inhibit various inflammatory pathways responsible for atherosclerosis. The exact mechanism is unknown, but beneficial effects on lipoprotein functions and on macrophage cholesterol metabolism have been described [88]. A recent meta-analysis of 28 observational studies suggests that the risk of cardiovascular events can be decreased by the use of TNF-inhibitors (RR 0.7; 95 %-CI 0.5-0.9; p = 0.005) and methotrexate (RR 0.7; 95 %-CI 0.6-0.9; p = 0.007) [25].

With regard to osteoporosis it has been suggested that TNF-inhibitors prevent further generalized bone loss by inhibiting bone resorption [89]. However, in most of these short-term and open-label trials, TNF-inhibitors were combined with methotrexate. Therefore, it needs to be determined whether this protective effect can be attributed to use of TNF-inhibitors by itself or the use of combination therapy and hence better RA disease control. In addition, no fracture data are currently available [89]. Interestingly, in early RA, short-term use of glucocorticoids may have a positive effect on BMD, due to its strong anti-inflammatory effects [90]. In a randomized, placebo-controlled, double-blind 2-year study by van der Goes et al., addition of 10 mg prednisone daily to a methotrexate-based tight control strategy did not result in a negative effect on BMD in early RA patients on bisphosphonates [90].

Few studies have prospectively examined the impact of anti-rheumatic treatment on body composition [91–95]. A small-sized randomised study of 21 months duration by Engvall et al. including 40 patients, the use of TNF-inhibitors was associated with an increase in body fat mass (+3.8 (1.6-5.9) kg in the TNF inhibitor group vs +0.4 (−1.5-2.2) kg (p = 0.04) in the conventional synthetic DMARD group). There were no changes in muscle mass or lipid profile [92]. Other studies with a shorter follow-up duration failed to show a change in body composition [93–95]. It needs to be determined whether these possible changes in body composition can be confirmed in other studies and if so, whether they are associated with development of cardiovascular disease on the long term.

In a recent systematic review and meta-analysis, the effect of TNF-inhibitors on depression and anxiety were evaluated [96]. Overall, effects were small or not significant. However, many studies have shown that anti-rheumatic therapy improves important patient reported outcomes including general well-being, fatigue and quality of life [97].

### Does treatment of comorbidities improve RA?

There is some evidence that medication regularly prescribed for comorbidities, such as statins, might also improve RA disease activity measures and lower inflammatory markers [98, 99]. In addition to their lipid-lowering effects, statins also exert an anti-inflammatory function, which is held responsible for the beneficial effect on RA disease activity. In the randomised, placebo-controlled Trial of Atorvastatin in Rheumatoid Arthritis (TARA), it was found that addition of atorvastatin to standard antirheumatic therapy significantly improved the DAS28 as compared to placebo (treatment group: −0.50, 95 %-CI −0.8 to −0.3; placebo group: +0.03, 95 %-CI −0.2 to 0.3) [99]. In a recent cohort study by Schoenfeld et al., it was concluded that statin use was independently associated with a 21 % lower risk of all-cause mortality among patients with RA (HR  0.8, 95 %-CI 0.7-0.9) [99].

There is limited evidence that denosumab, a human monoclonal antibody against the Receptor activator of nuclear factor kappa B ligand and used in the treatment of osteoporosis, may inhibit the development of joint erosions in patients with RA [100, 101]. However, denosumab had no effect on joint space narrowing or on RA disease activity [101].

Although selective serotonin reuptake inhibitors have been reported to exibit anti-inflammatory effects in addition to their antidepressant effects, there is currently insufficient evidence that treatment of depression positively or negatively influences RA disease-specific outcome measures and other clinical outcomes [102–104]. In addition, the evidence to routinely prescribe antidepressants as analgesics in patients with RA is also inconclusive [105].

## Conclusions

The research to date has successfully identified the epidemiology of comorbidity in patients with RA, a variety of determinants that influence outcome and to some extent the consequences associated with the presence of comorbidity. However, the magnitude of effect of ageing and comorbidity on outcome measures and RA management is largely unknown. Moreover, for many of the comorbidities, it is equally unclear whether they should be managed similarly in middle aged versus older patients. It seems clear that elderly RA patients who also face comorbidity will need a different management approach since the needs of these patients are more than just the sum of needs in relation to single diseases [106]. The symptoms of RA and comorbidities may be overlapping, treatments may interact, underlying pathophysiology may be shared and the course of all diseases may be altered. As a consequence, the current RA treatment strategies might not be directly translatable to elderly patients with RA and comorbidity [107]. Research should focus on the impact of comorbidities on screening, diagnosis and outcome measurement of patients with RA. Nowadays, elderly patients with comorbidities are often excluded from intervention studies [108]. Future clinical trials should however take the complex treatment-reality of these patients into consideration by developing for instance comprehensive comorbidity measures in order to correct for confounding and effect modification in clinical trials [109, 110]. This may ultimately result in the development of recommendations that can guide the complex management decisions that need to be made in the case of an ageing RA patient who faces comorbidity. In doing so, a goal-oriented approach should be prioritized above a disease-centered approach. Maintaining maximal functional status and active social participation are essential components of a goal-oriented approach. Avoiding inefficient healthcare utilisation and medication side-effects (e.g. suffering more from the treatment than from the disease) is important [111].

We propose the following priority clinical research areas:

- Improve our understanding on the role of RA and its management on ageing and occurrence as well as course of comorbidity.
- Explore barriers within patients and healthcare providers with regard to (1) prioritization of health issues and (2) executing a realistic care plan when dealing with comorbidity;
- Adjusting general and RA-specific outcome measures that account for ageing and comorbidity;
- Develop, evaluate and implement models for integrated, coordinated and goal-oriented care.

### Availability of data and materials

Data available from published papers as per references.

## Abbreviations

ACPA: anti-citrullinated protein antibodies
ACR: American College of Rheumatology
BMD: Bone Mass Density
BMI: Body Mass Index
CDAI: Clinical Disease Activity Index
CI: Confidence Interval
CRP: C-reactive Protein
CVD: cardiovascular disease
DMARD: Disease Modifying Anti-Rheumatic Drug
ESR: Erythrocyte Sedimentation Rate
EULAR: European League Against Rheumatism
HAQ: Health Assessment Questionnaire
HR: Hazard Ratio
LBM: lean body mass
OR: Odds Ratio
RA: rheumatoid arthritis
RCT: Randomised Controlled Trial
RR: Relative Risk
SIR: Standardized Incidence Ratio
TNF: Tumor Necrosis Factor
VAS: Visual Analogue Scale

## References

[1] Project Europe 2030. http://espas.eu/orbis/sites/default/files/generated/document/en/Project%20Europe%202030.pdf. Accessed 30 December 2015.

[2] Cross M, Smith E, Hoy D, Carmona L, Wolfe F, Vos T, Williams B (2014). The global burden of rheumatoid arthritis: estimates from the global burden of disease 2010 study. Ann Rheum Dis.

[3] Fautrel B, Verstappen SM, Boonen A (2011). Economic consequences and potential benefits. Best Pract Res Clin Rheumatol.

[4] Gavan S, Harrison M, Iglesias C, Barton A, Manca A, Payne K (2014). Economics of stratified medicine in rheumatoid arthritis. Curr Rheumatol Rep.

[5] Gullick NJ, Scott DL (2011). Co-morbidities in established rheumatoid arthritis. Best Pract Res Clin Rheumatol.

[6] Dougados M, Soubrier M, Antunez A, Balint P, Balsa A, Buch MH (2014). Prevalence of comorbidities in rheumatoid arthritis and evaluation of their monitoring: results of an international, cross-sectional study (COMORA). Ann Rheum Dis.

[7] Gabriel SE (2008). Why do people with rheumatoid arthritis still die prematurely?. Ann Rheum Dis.

[8] Gabriel S, Michaud K (2009). Epidemiological studies in incidence, prevalence, mortality, and co-morbidity of the rheumatic diseases. Arthritis Res Ther.

[9] Michaud K, Wolfe F (2007). Comorbidities in rheumatoid arthritis. Best Pract Res Clin Rheumatol.

[10] Dougados M, Soubrier M, Perrodeau E, Gossec L, Fayet F, Gilson M (2015). Impact of a nurse-led programme on comorbidity management and impact of a patient self-assessment of disease activity on the management of rheumatoid arthritis: results of a prospective, multicentre, randomised, controlled trial (COMEDRA). Ann Rheum Dis.

[11] MacLean CH, Louie R, Leake B, McCaffrey DF, Paulus HE, Brook RH (2000). Quality of care for patients with rheumatoid arthritis. JAMA.

[12] Goronzy JJ, Weyand CM (2003). Aging, autoimmunity and arthritis: T-cell senescence and contraction of T-cell repertoire diversity - catalysts of autoimmunity and chronic inflammation. Arthritis Res Ther.

[13] Boots AM, Maier AB, Stinissen P, Masson P, Lories RJ, De Keyser F (2013). The influence of ageing on the development and management of rheumatoid arthritis. Nat Rev Rheumatol.

[14] Goronzy JJ, Weyand CM (2013). Understanding immunosenescence to improve responses to vaccines. Nat Immunol.

[15] Weng NP (2006). Aging of the immune system: how much can the adaptive immune system adapt?. Immunity.

[16] Goronzy JJ, Li G, Yang Z, Weyand CM (2013). The janus head of T cell aging - autoimmunity and immunodeficiency. Front Immunol.

[17] Straub RH, Schölmerich J, Cutolo M (2003). The multiple facets of premature aging in rheumatoid arthritis. Arthritis Rheum.

[18] Michaud M, Balardy L, Moulis G, Gaudin C, Peyrot C, Vellas B (2013). Proinflammatory cytokines, aging, and age-related diseases. J Am Med Dir Assoc.

[19] Solomon DH, Karlson EW, Rimm EB, Cannuscio CC, Mandl LA, Manson JE (2003). Cardiovascular morbidity and mortality in women diagnosed with rheumatoid arthritis. Circulation.

[20] Avina-Zubieta JA, Thomas J, Sadatsafavi M, Lehman AJ, Lacaille D (2012). Risk of incident cardiovascular events in patients with rheumatoid arthritis: a meta-analysis of observational studies. Ann Rheum Dis.

[21] Meune C, Touzé E, Trinquart L, Allanore Y (2009). Trends in cardiovascular mortality in patients with rheumatoid arthritis over 50 years: a systematic review and meta-analysis of cohort studies. Rheumatology (Oxford).

[22] Baghdadi LR, Woodman RJ, Shanahan EM, Mangoni AA (2015). The impact of traditional cardiovascular risk factors on cardiovascular outcomes in patients with rheumatoid arthritis: a systematic review and meta-analysis. PLoS One.

[23] John H, Kitas G (2012). Inflammatory arthritis as a novel risk factor for cardiovascular disease. Eur J Intern Med.

[24] Nurmohamed MT, Heslinga M, Kitas GD (2015). Cardiovascular comorbidity in rheumatic diseases. Nat Rev Rheumatol.

[25] Roubille C, Richer V, Starnino T, McCourt C, McFarlane A, Fleming P (2015). The effects of tumour necrosis factor inhibitors, methotrexate, non-steroidal anti-inflammatory drugs and corticosteroids on cardiovascular events in rheumatoid arthritis, psoriasis and psoriatic arthritis: a systematic review and meta-analysis. Ann Rheum Dis.

[26] Lindhardsen J, Ahlehoff O, Gislason GH, Madsen OR, Olesen JB, Torp-Pedersen C (2011). The risk of myocardial infarction in rheumatoid arthritis and diabetes mellitus: a Danish nationwide cohort study. Ann Rheum Dis.

[27] Lake F, Proudman S (2014). Rheumatoid arthritis and lung disease: from mechanisms to a practical approach. Semin Respir Crit Care Med.

[28] Bongartz T, Nannini C, Medina-Velasquez YF, Achenbach SJ, Crowson CS, Ryu JH (2010). Incidence and mortality of interstitial lung disease in rheumatoid arthritis: a population-based study. Arthritis Rheum.

[29] Ungprasert P, Srivali N, Cheungpasitporn W, Davis Iii JM. Risk of incident chronic obstructive pulmonary disease in patients with rheumatoid arthritis: A systematic review and meta-analysis. Joint Bone Spine. 2015. doi: 10.1016/j.jbspin.2015.05.016. [Epub ahead of print]. 10.1016/j.jbspin.2015.05.016 26709254

[30] Smitten AL, Simon TA, Hochberg MC, Suissa S (2008). A meta-analysis of the incidence of malignancy in adult patients with rheumatoid arthritis. Arthritis Res Ther.

[31] Baecklund E, Iliadou A, Askling J, Ekbom A, Backlin C, Granath F (2006). Association of chronic inflammation, not its treatment, with increased lymphoma risk in rheumatoid arthritis. Arthritis Rheum.

[32] Turesson C, Matteson EL (2013). Malignancy as a comorbidity in rheumatic diseases. Rheumatology (Oxford).

[33] Parikh-Patel A, White RH, Allen M, Cress R (2009). Risk of cancer among rheumatoid arthritis patients in California. Cancer Causes Control.

[34] Ramiro S, Gaujoux-Viala C, Nam JL, Smolen JS, Buch M, Gossec L (2014). Safety of synthetic and biological DMARDs: a systematic literature review informing the 2013 update of the EULAR recommendations for management of rheumatoid arthritis. Ann Rheum Dis.

[35] Rachner TD, Khosla S, Hofbauer LC (2011). Osteoporosis: now and the future. Lancet.

[36] Van Staa TP, Geusens P, Bijlsma JW, Leufkens HG, Cooper C (2006). Clinical assessment of the long-term risk of fracture in patients with rheumatoid arthritis. Arthritis Rheum.

[37] Rajbhandary R, Khezri A, Panush RS (2011). Rheumatoid cachexia: what is it and why is it important?. J Rheumatol.

[38] Muscaritoli M, Anker SD, Argilés J, Aversa Z, Bauer JM, Biolo G (2010). Consensus definition of sarcopenia, cachexia and pre-cachexia: joint document elaborated by Special Interest Groups (SIG) "cachexia-anorexia in chronic wasting diseases" and "nutrition in geriatrics". Clin Nutr.

[39] El Maghraoui A, Sadni S, Rezqi A, Bezza A, Achemlal L, Mounach A (2015). Does Rheumatoid Cachexia Predispose Patients with Rheumatoid Arthritis to Osteoporosis and Vertebral Fractures?. J Rheumatol.

[40] Summers GD, Metsios GS, Stavropoulos-Kalinoglou A, Kitas GD (2010). Rheumatoid cachexia and cardiovascular disease. Nat Rev Rheumatol.

[41] Verschueren S, Gielen E, O'Neill TW, Pye SR, Adams JE, Ward KA (2013). Sarcopenia and its relationship with bone mineral density in middle-aged and elderly European men. Osteoporos Int.

[42] He H, Liu Y, Tian Q, Papasian CJ, Hu T, Deng HW (2016). Relationship of sarcopenia and body composition with osteoporosis. Osteoporos Int.

[43] Appenzeller S, Bertolo MB, Costallat LT (2004). Cognitive impairment in rheumatoid arthritis. Methods Find Exp Clin Pharmacol.

[44] Bartolini M, Candela M, Brugni M, Catena L, Mari F, Pomponio G (2002). Are behaviour and motor performances of rheumatoid arthritis patients influenced by subclinical cognitive impairments? A clinical and neuroimaging study. Clin Exp Rheumatol.

[45] Shin SY, Katz P, Wallhagen M, Julian L (2012). Cognitive impairment in persons with rheumatoid arthritis. Arthritis Care Res (Hoboken).

[46] Shin SY, Julian L, Katz P (2013). The relationship between cognitive function and physical function in rheumatoid arthritis. J Rheumatol.

[47] Wallin K, Solomon A, Kåreholt I, Tuomilehto J, Soininen H, Kivipelto M (2012). Midlife rheumatoid arthritis increases the risk of cognitive impairment two decades later: a population-based study. J Alzheimers Dis.

[48] Brown SC, Glass JM, Park DC (2002). The relationship of pain and depression to cognitive function in rheumatoid arthritis patients. Pain.

[49] Joaquim AF, Appenzeller S. Neuropsychiatric manifestations in rheumatoid arthritis. Autoimmun Rev 2015. doi:10.1016/j.autrev.2015.07.015. 10.1016/j.autrev.2015.07.015 26238502

[50] Kessler RC, Berglund P, Demler O, Jin R, Koretz D, Merikangas KR (2003). National Comorbidity Survey Replication. The epidemiology of major depressive disorder: results from the National Comorbidity Survey Replication (NCS-R). JAMA.

[51] Kessler RC, Chiu WT, Demler O, Merikangas KR, Walters EE (2005). Prevalence, severity, and comorbidity of 12-month DSM-IV disorders in the National Comorbidity Survey Replication. Arch Gen Psychiatry.

[52] Matcham F, Rayner L, Steer S, Hotopf M (2013). The prevalence of depression in rheumatoid arthritis: a systematic review and meta-analysis. Rheumatology (Oxford).

[53] Covic T, Cumming SR, Pallant JF, Manolios N, Emery P, Conaghan PG, Tennant A (2012). Depression and anxiety in patients with rheumatoid arthritis: prevalence rates based on a comparison of the Depression, Anxiety and Stress Scale (DASS) and the hospital, Anxiety and Depression Scale (HADS). BMC Psychiatry.

[54] Lok EY, Mok CC, Cheng CW, Cheung EF (2010). Prevalence and determinants of psychiatric disorders in patients with rheumatoid arthritis. Psychosomatics.

[55] Van der Heijde DMFM, van 't Hof MA, van Riel PLCM, Theunisse LAM, Lubberts EW, van Leeuwen MA (1990). Judging disease activity in clinical practice in rheumatoid arthritis: first step in the development of a disase activity score. Ann Rheum Dis.

[56] Fries JF, Spitz P, Kraines RG, Holman HR (1980). Measurement of patient outcome in arthritis. Arthritis Rheum.

[57] Pinals RS, Masi AT, Larsen RA (1981). Preliminary criteria for clinical remission in rheumatoid arthritis. Arthritis Rheum.

[58] Ranganath VK, Maranian P, Elashoff DA, Woodworth T, Khanna D, Hahn T (2013). Comorbidities are associated with poorer outcomes in community patients with rheumatoid arthritis. Rheumatology (Oxford).

[59] Arnold MB, Bykerk VP, Boire G, Haraoui BP, Hitchon C, Thorne C, CATCH Investigators (2014). Are there differences between young- and older-onset early inflammatory arthritis and do these impact outcomes? An analysis from the CATCH cohort. Rheumatology (Oxford).

[60] Zink A, Strangfeld A, Schneider M, Herzer P, Hierse F, Stoyanova-Scholz M (2006). Effectiveness of tumor necrosis factor inhibitors in rheumatoid arthritis in an observational cohort study: comparison of patients according to their eligibility for major randomized clinical trials. Arthritis Rheum.

[61] Burmester GR, Ferraccioli G, Flipo RM, Monteagudo-Sáez I, Unnebrink K, Kary S (2008). Clinical remission and/or minimal disease activity in patients receiving adalimumab treatment in a multinational, open-label, twelve-week study. Arthritis Rheum.

[62] Sokka T, Hetland ML, Mäkinen H, Kautiainen H, Hørslev-Petersen K, Luukkainen RK (2008). Questionnaires in Standard Monitoring of Patients With Rheumatoid Arthritis Group. Remission and rheumatoid arthritis: data on patients receiving usual care in twenty-four countries. Arthritis Rheum.

[63] Krishnan E, Häkkinen A, Sokka T, Hannonen P (2005). Impact of age and comorbidities on the criteria for remission and response in rheumatoid arthritis. Ann Rheum Dis.

[64] Sokka T, Mäkinen H, Hannonen P, Pincus T (2007). Most people over age 50 in the general population do not meet ACR remission criteria or OMERACT minimal disease activity criteria for rheumatoid arthritis. Rheumatology (Oxford).

[65] Toms J, Soukup T, Bradna P, Hrncir Z (2010). Disease activity composite indices in patients with rheumatoid arthritis and concomitant fibromyalgia. J Rheumatol.

[66] Radner H, Smolen JS, Aletaha D (2011). Comorbidity affects all domains of physical function and quality of life in patients with rheumatoid arthritis. Rheumatology (Oxford).

[67] Stoffer MA, Schoels MM, Smolen JS, et al. Evidence for treating rheumatoid arthritis to target: results of a systematic literature search update. Ann Rheum Dis. 2015:doi: 10.1136/annrheumdis-2015-207526. [Epub ahead of print]. 10.1136/annrheumdis-2015-207526 PMC4717391 25990290

[68] Kremers HM, Nicola P, Crowson CS, O'Fallon WM, Gabriel SE (2004). Therapeutic strategies in rheumatoid arthritis over a 40-year period. J Rheumatol.

[69] Fraenkel L, Rabidou N, Dhar R (2006). Are rheumatologists' treatment decisions influenced by patients' age?. Rheumatology (Oxford).

[70] Tutuncu Z, Reed G, Kremer J, Kavanaugh A (2006). Do patients with older-onset rheumatoid arthritis receive less aggressive treatment?. Ann Rheum Dis.

[71] Roubille C, Richer V, Starnino T, McCourt C, McFarlane A, Fleming P (2015). Evidence-based Recommendations for the Management of Comorbidities in Rheumatoid Arthritis, Psoriasis, and Psoriatic Arthritis: Expert Opinion of the Canadian Dermatology-Rheumatology Comorbidity Initiative. J Rheumatol.

[72] Leitzmann MF, Park Y, Blair A, Ballard-Barbash R, Mouw T, Hollenbeck AR, Schatzkin A (2007). Physical activity recommendations and decreased risk of mortality. Arch Intern Med.

[73] Berrington de Gonzalez A, Hartge P, Cerhan JR (2010). Body-mass index and mortality among 1.46 million white adults. N Engl J Med.

[74] Sugiyama D, Nishimura K, Tamaki K, Tsuji G, Nakazawa T, Morinobu A, Kumagai S (2010). Impact of smoking as a risk factor for developing rheumatoid arthritis: a meta-analysis of observational studies. Ann Rheum Dis.

[75] Källberg H, Ding B, Padyukov L, Bengtsson C, Rönnelid J, Klareskog L (2011). EIRA Study Group. Smoking is a major preventable risk factor for rheumatoid arthritis: estimations of risks after various exposures to cigarette smoke. Ann Rheum Dis.

[76] Mattey DL, Hutchinson D, Dawes PT, Nixon NB, Clarke S, Fisher J (2002). Smoking and disease severity in rheumatoid arthritis: association with polymorphism at the glutathione S-transferase M1 locus. Arthritis Rheum.

[77] Finckh A, Dehler S, Costenbader KH, Gabay C, Swiss Clinical Quality Management project for RA (2007). Cigarette smoking and radiographic progression in rheumatoid arthritis. Ann Rheum Dis.

[78] De Rooy DP, van Nies JA, Kapetanovic MC, Kristjansdottir H, Andersson ML, Forslind K (2014). Smoking as a risk factor for the radiological severity of rheumatoid arthritis: a study on six cohorts. Ann Rheum Dis.

[79] Metsios GS, Stavropoulos-Kalinoglou A, Veldhuijzen van Zanten JJ, Treharne GJ, Panoulas VF (2008). Rheumatoid arthritis, cardiovascular disease and physical exercise: a systematic review. Rheumatology (Oxford).

[80] Hurkmans E, van der Giesen FJ, Vliet Vlieland TP, Schoones J, Van den Ende EC. Dynamic exercise programs (aerobic capacity and/or muscle strength training) in patients with rheumatoid arthritis. Cochrane Database Syst Rev. 2009;CD006853. 10.1002/14651858.CD006853.pub2 PMC6769170 19821388

[81] Plasqui G (2008). The role of physical activity in rheumatoid arthritis. Physiol Behav.

[82] Cooney JK, Law RJ, Matschke V, Lemmey AB, Moore JP, Ahmad Y (2011). Benefits of exercise in rheumatoid arthritis. J Aging Res.

[83] Veldhuijzen van Zanten JJ, Rouse PC, Hale ED, Ntoumanis N, Metsios GS, Duda JL, et al. Perceived Barriers, Facilitators and Benefits for Regular Physical Activity and Exercise in Patients with Rheumatoid Arthritis: A Review of the Literature. Sports Med 2015 Jul 29. [Epub ahead of print] 10.1007/s40279-015-0363-2 PMC4579262 26219268

[84] Ajeganova S, Andersson ML, Hafström I, BARFOT Study Group (2013). Association of obesity with worse disease severity in rheumatoid arthritis as well as with comorbidities: a long-term followup from disease onset. Arthritis Care Res (Hoboken).

[85] Papadakis JA, Sidiropoulos PI, Karvounaris SA, Vrentzos GE, Spanakis EK, Ganotakis ES, Kritikos HD (2007). Metabolic syndrome is common among middle-to-older aged Mediterranean patients with rheumatoid arthritis and correlates with disease activity: a retrospective, cross-sectional, controlled, study. Ann Rheum Dis.

[86] Van der Helm-van Mil AH, van der Kooij SM, Allaart CF, Toes RE, Huizinga TW (2008). A high body mass index has a protective effect on the amount of joint destruction in small joints in early rheumatoid arthritis. Ann Rheum Dis.

[87] Peters MJ, Symmons DP, McCarey D, Dijkmans BA, Nicola P, Kvien TK (2010). EULAR evidence-based recommendations for cardiovascular risk management in patients with rheumatoid arthritis and other forms of inflammatory arthritis. Ann Rheum Dis.

[88] Ronda N, Greco D, Adorni MP, Zimetti F, Favari E, Hjeltnes G (2015). Newly identified antiatherosclerotic activity of methotrexate and adalimumab: complementary effects on lipoprotein function and macrophage cholesterol metabolism. Arthritis Rheumatol.

[89] Sakthiswary R, Das S (2013). The effects of TNF α antagonist therapy on bone metabolism in rheumatoid arthritis: a systematic review. Curr Drug Targets.

[90] Van der Goes MC, Jacobs JW, Jurgens MS, Bakker MF, van der Veen MJ, van der Werf JH (2013). Are changes in bone mineral density different between groups of early rheumatoid arthritis patients treated according to a tight control strategy with or without prednisone if osteoporosis prophylaxis is applied?. Osteoporos Int..

[91] Toussirot É (2015). Effects of TNFα inhibitors on adiposity and other cardiovascular risk factors: implications for the cardiovascular prognosis in patients with rheumatoid arthritis. Expert Opin Drug Saf.

[92] Engvall IL, Tengstrand B, Brismar K, Hafström I (2010). Infliximab therapy increases body fat mass in early rheumatoid arthritis independently of changes in disease activity and levels of leptin and adiponectin: a randomised study over 21 months. Arthritis Res Ther.

[93] Metsios GS, Stavropoulos-Kalinoglou A, Douglas KM, Koutedakis Y, Nevill AM, Panoulas VF (2007). Blockade of tumour necrosis factor-alpha in rheumatoid arthritis: effects on components of rheumatoid cachexia. Rheumatology (Oxford).

[94] Marcora SM, Chester KR, Mittal G, Lemmey AB, Maddison PJ (2006). Randomized phase 2 trial of anti-tumor necrosis factor therapy for cachexia in patients with early rheumatoid arthritis. Am J Clin Nutr.

[95] Serelis J, Kontogianni MD, Katsiougiannis S, Bletsa M, Tektonidou MG, Skopouli FN (2008). Effect of anti-TNF treatment on body composition and serum adiponectin levels of women with rheumatoid arthritis. Clin Rheumatol.

[96] Abbott R, Whear R, Nikolaou V, Bethel A, Coon JT, Stein K (2015). Tumour necrosis factor-α inhibitor therapy in chronic physical illness: A systematic review and meta-analysis of the effect on depression and anxiety. J Psychosom Res.

[97] Kekow J, Moots R, Khandker R, Melin J, Freundlich B, Singh A (2011). Improvements in patient-reported outcomes, symptoms of depression and anxiety, and their association with clinical remission among patients with moderate-to-severe active early rheumatoid arthritis. Rheumatology (Oxford).

[98] McCarey DW, McInnes IB, Madhok R, Hampson R, Scherbakov O, Ford I (2004). Trial of Atorvastatin in Rheumatoid Arthritis (TARA): double-blind, randomised placebo-controlled trial. Lancet.

[99] Schoenfeld SR, Lu L, Rai SK, Seeger JD, Zhang Y, Choi HK. Statin use and mortality in rheumatoid arthritis: a general population-based cohort study. Ann Rheum Dis 2015. doi: 10.1136/annrheumdis-2015-207714. 10.1136/annrheumdis-2015-207714 PMC5399680 26245753

[100] Ferrari-Lacraz S, Ferrari S (2011). Do RANKL inhibitors (denosumab) affect inflammation and immunity?. Osteoporos Int.

[101] Cohen SB, Dore RK, Lane NE (2008). Denosumab Rheumatoid Arthritis Study Group. Denosumab treatment effects on structural damage, bone mineral density, and bone turnover in rheumatoid arthritis: a twelve-month, multicenter, randomized, double-blind, placebo-controlled, phase II clinical trial. Arthritis Rheum.

[102] Sacre S, Medghalchi M, Gregory B, Brennan F, Williams R (2010). Fluoxetine and citalopram exhibit potent antiinflammatory activity in human and murine models of rheumatoid arthritis and inhibit toll-like receptors. Arthritis Rheum.

[103] Bawa FL, Mercer SW, Atherton RJ, Clague F, Keen A, Scott NW (2015). Does mindfulness improve outcomes in patients with chronic pain? Systematic review and meta-analysis. Br J Gen Pract.

[104] Richards BL, Whittle SL, Buchbinder R. Antidepressants for pain management in rheumatoid arthritis. Cochrane Database Syst Rev. 2011:CD008920. 10.1002/14651858.CD008920.pub2 PMC12214451 22071859

[105] Richards BL, Whittle SL, van der Heijde DM, Buchbinder R (2012). The efficacy and safety of antidepressants in inflammatory arthritis: a Cochrane systematic review. J Rheumatol Suppl.

[106] Barnett K, Mercer SW, Norbury M, Watt G, Wyke S, Guthrie B (2012). Epidemiology of multimorbidity and implications for health care, research, and medical education: a cross-sectional study. Lancet.

[107] Loza E, Lajas C, Andreu JL, Balsa A, González-Álvaro I, Illera O (2015). Consensus statement on a framework for the management of comorbidity and extra-articular manifestations in rheumatoid arthritis. Rheumatol Int.

[108] Konrat C, Boutron I, Trinquart L, Auleley GR, Ricordeau P, Ravaud P (2012). Underrepresentation of elderly people in randomised controlled trials. The example of trials of 4 widely prescribed drugs. PLoS One.

[109] De Groot V, Beckerman H, Lankhorst GJ, Bouter LM (2003). How to measure comorbidity. a critical review of available methods. J Clin Epidemiol.

[110] El Miedany Y (2015). Co-morbidity index in rheumatoid arthritis: time to think. Clin Rheumatol.

[111] Mold J, Blake G, Becker L (1991). Goal-oriented medical care. Fam Med.

